# Osteoarticular infections in children in Qatar: A retrospective study

**DOI:** 10.5339/qmj.2025.69

**Published:** 2025-08-20

**Authors:** Mohamed Elkalaf, Shabina Khan, Maisaa Elzain, Eiman Hamid, Saira Shehzad, Khalid Al-Kharraz, Tawa Olukade, Mohammed Alkuwari, Mohammed Al Amri, Ashraf Soliman, Sahnoun Ahmed Khalil

**Affiliations:** 1 Section of Academic General Pediatrics, Department of Pediatrics, Hamad General Hospital, Doha, Qatar; 2 Department of Pediatrics, Hamad General Hospital, Doha, Qatar; 3 Section of Pediatric Clinical Pharmacy, Pharmacy Department, Hamad General Hospital, Doha, Qatar *Email: akhalil7@hamad.qa

**Keywords:** Osteoarticular, osteomyelitis, pediatrics, septic arthritis, State of Qatar

## Abstract

**Background:**

Osteoarticular infections (OAIs) in Qatar’s pediatric population represent a significant source of morbidity, highlighting the need for a comprehensive evaluation of their clinical, microbiological, and treatment characteristics. Understanding the presentation and management of these infections is essential for optimizing patient outcomes and guiding future therapeutic approaches.

**Methods:**

Retrospective cohort study of 62 hospitalized children with OAIs treated at Hamad Medical Corporation from 2016 to 2018.

**Results:**

Over 2 years, 62 patients were diagnosed and treated for OAIs. Osteomyelitis emerged as the most prevalent infection type, comprising 48.4% of cases, followed by septic arthritis at 20.9% and concurrent infections at 17.7%. The cohort demonstrated a male predominance of 66.1%. The common clinical manifestations included fever (77.4%), functional limitations (82.3%), and pain (88.7%). *Staphylococcus aureus* was the most frequently isolated pathogen, present in 24.1% (15/62) of cases, with approximately 50% of these being methicillin-resistant *Staphylococcus aureus*. Among the radiological studies conducted, 75% revealed abnormal ultrasound findings, while all patients exhibited abnormal magnetic resonance imaging results (*n* = 53). The antibiotic regimen most frequently prescribed was clindamycin (79%), followed by the cephalosporin ceftriaxone (67.7%). The group with concurrent infections showed the longest duration of both parenteral and oral antibiotic therapy, the highest complication rates, and the longest median hospital stay of 21 days.

**Conclusions:**

OAIs present substantial clinical challenges, marked by complex presentations and diagnostic difficulties. *Staphylococcus aureus* remains the predominant pathogen, with methicillin-resistant *Staphylococcus aureus* accounting for nearly half of the cases. Concurrent infections are linked with more severe complications and prolonged hospitalizations, though they also exhibit the highest rates of positive microbiological results.

## INTRODUCTION

Osteoarticular infections (OAIs) continue to be a significant source of morbidity in pediatric populations. These infections can present as septic arthritis (SA), osteomyelitis (OM), or a combination of both, depending on their anatomical location. SA is characterized by joint inflammation resulting from infectious etiology, which may include bacterial, fungal, mycobacterial, viral, or other pathogens. In contrast, OM involves inflammation of the bone and bone marrow due to microbial infection. Acute OAIs are predominantly bacterial; however, other etiologies must also be considered.^1,2^

OAIs have an estimated annual incidence of 1 to 13 per 100,000 children in developed countries, whereas in developing countries, the incidence can be as high as 200 per 100,000 children.^3^ In pediatric populations, the primary route of infection for OAIs starts through the spread of infection from the bloodstream to the bones and/ or joints. However, infections can also begin from direct introduction or the spread of infection from nearby tissues.^2^ The incidence of OM and SA in children shows notable geographic variation. Globally, the incidence of SA in children is reported to range from 1 to 37 cases per 100,000, while OM affects approximately 1.2 to 13 cases per 100,000 children annually.^3,4^ Some countries have noted a decrease in the incidence over time,^5,6^ whereas others, including the United States, have noted an increase.^6–8^ Whether this variation is due to emerging resistant organisms, local changes in antibiotic patterns, improved diagnostic methods, or new antibiotics still needs to be determined. Timely diagnosis and intervention are imperative in managing pediatric OAIs, as delays can lead to severe and often irreversible sequelae. These include longitudinal growth arrest, joint stiffness, angular deformities, and chronic infections. Additionally, complications such as avascular necrosis, sepsis, multiorgan failure, and death may arise from delayed treatment, underscoring the critical need for prompt medical attention.^9,10^

In pediatric OM, *Staphylococcus aureus* (*S. aureus*) is identified as the most prevalent pathogen, with *Streptococcus pyogenes* as the next most common.^11,12^ In recent years, the incidence of community-acquired methicillin-resistant *S. aureus* (CA-MRSA) has risen among hospitalized children with pediatric OAIs, particularly in the United States.13–16 In contrast, the prevalence of methicillin-resistant *Staphylococcus aureus* (MRSA) remains comparatively low among European children with bone and joint infections.^17,18^ The introduction of pneumococcal conjugate vaccines (PCV7 and PCV13) and the *Haemophilus influenzae* type b vaccine into the childhood immunization schedule has significantly reduced the incidence of invasive diseases caused by these pathogens.^19–22^ Recently, *Kingella kingae* (*K. kingae*) has emerged as a prominent pathogen in pediatric OAIs, with detection rates reaching up to 82% in children under 5 years of age with SA.^23,24^

In Qatar, there are no national studies specifically addressing OAIs in pediatric populations. However, a previous national study conducted between 2000 and 2013 investigated the epidemiology, clinical presentation, and microbiology of OM alone.^25^ Given the close association between OM and SA, our objective was to undertake a comprehensive descriptive study that includes both types of OAIs. This study aims to explore the epidemiology, etiology, clinical presentation, diagnostic investigations, treatment outcomes, and complications associated with these infections.

## METHODS

### Study design, setting, and participants

This retrospective cohort study focused on hospitalized children with OAIs treated from 2016 to 2018. Patients were identified through *International Classification of Diseases-Tenth Revision* codes used for diagnosing OM and SA, with diagnoses confirmed via chart reviews. The study was primarily conducted at Hamad Medical Corporation (HMC) in Qatar, a cosmopolitan hub with a diverse patient population.

Inclusion criteria encompassed all pediatric cases aged over 1 month to 14 years diagnosed with OM, SA, or concurrent infections. Exclusion criteria included neonates and immunodeficient patients. Out of 152 records retrieved, 68 were duplicates, and 22 were excluded (seven with immunodeficiency, four with soft tissue infections such as abscess and bursitis, four with rheumatologic conditions including Chronic Recurrent Multifocal Osteomyelitis and Juvenile idiopathic arthritis, three with other infections like sinusitis, mastoiditis, and mediastinitis, two with bacteremia only, one with a bone tumor, and one with complications from a previous bone infection). Ultimately, 62 cases were included in this review. For comparison, cases were categorized into isolated SA, various types of OM (acute, subacute, and chronic), and concurrent infections involving both SA and OM. The identification and susceptibility profiling of all bacteria from positive cultures were determined by using an automated susceptibility system, VITEK 2 (bioMérieux, Marcy-l’Étoile, France), according to the Clinical and Laboratory Standards Institute guidelines.

### Data collection and study variables

We retrieved data relating to patients’ demographics, past medical history, treatment given, and laboratory and radiology data related to this study objective from electronic medical records. The diagnosis of OAIs is confirmed by histopathologic evidence of either inflammation in a surgical specimen of bone, a positive culture or Gram stain in an aspirate or biopsy of bone, or a periosteal fluid collection. In practice, however, and in our center, patient workup involves the combination of clinical features, imaging, microbiologic features, and the response to empiric antimicrobial therapy.

Data were extracted from the laboratory, microbiology, and electronic medical records (Cerner). Collected data were documented in a data collection sheet, which included variables on demographic data with nationality as Qatari and non-Qatari, clinical presentation, laboratory results, imaging studies, used medications, and complications. Data were initially collected using a protected data sheet, securely stored and accessible only by the primary investigator. Subsequently, the data were de-identified, entered into an Excel sheet, and analyzed exclusively on a dedicated laptop under the primary investigator’s control for publication purposes.

### Data analysis

Descriptive statistics were used to summarize the data using numbers and percentages, mean and standard deviation, or median and interquartile range (IQR) as applicable to the data distribution. Comparisons between all three groups were conducted for selected categorical or continuous variables using χ^2^ tests, ANOVA, or independent Kruskal-Wallis test as applicable. Statistical significance was set at *p* < 0.05. Statistical analysis was performed using IBM SPSS 27 statistical software.

## RESULTS

### Patient demographics and characteristics

We reviewed 62 children, of whom 66.1% (*n* = 41) were males and 37.1% (*n* = 23) were Qatari nationals.

The number of cases with available data for key variables was as follows: discharge C-reactive protein (CRP; 56), initial erythrocyte sedimentation rate (ESR; 57), discharge ESR (48), initial procalcitonin (19), discharge procalcitonin (14), magnetic resonance imaging (MRI; 53), ultrasound (51), X-ray (54), computed tomography (CT) scan (3), blood culture (59), aspirate culture (32), and tissue culture (27). This indicates that some data points were not recorded for all patients.

The overall mean age was 6.2 ± 3.5 years. OM was the most prevalent type of infection affecting (48.4%; *n* = 30) of the patients, followed by SA (20.9%; *n* = 21) and concurrent infections (17.7%; *n* = 11). Children with isolated SA were the youngest (5.4 ± 3.1 years), followed by children with OM (5.9 ± 3.5 years), while the oldest group was the concurrent infections at 8.3 ± 3.8 years (Table 1). More than one-third of the cases had a history of trauma. The majority presented with fever (77.4%), limitation of function (82.3%), and pain (88.7%). Additionally, about half exhibited other localized signs and symptoms (58.3%). The lower extremities were the most frequently affected sites in these children, accounting for 80.4% of cases, compared with 19.6% in the upper extremities. Within each group, the distribution of lower to upper extremities involvement was 82.4% versus 17.6% in SA, 79.2% versus 20.8% in OM, and 80% versus 20% in concurrent infections (Table 2).

### Hospitalization and laboratory parameters

The median length of stay (LOS) was similar between the SA and OM groups, but significantly longer in the concurrent infections group, with a median of 21 (IQR, 19–40) days, which was more than 10 days longer than the other groups (*p* = 0.002).

There was a marked but nonsignificant difference in the median initial CRP, which was highest in the concurrent infections (141; IQR, 44–249), followed by SA (102; IQR, 52–134), then the OM group (54; IQR, 22–111; *p* = 0.056; Table 3). The median initial procalcitonin levels showed a significant difference, being the highest in the concurrent infections group (*p* = 0.032), although initial procalcitonin data were available for only 19 patients. No significant differences were observed among the groups in initial and discharge ESR levels.

Upon admission, the median baseline white blood cell (WBC) count was 10.9 (IQR, 9.3–16.4), which decreased to a median of 7.9 (IQR, 6.8–9.1) at discharge. Despite this noticeable difference, statistical analysis indicated it was not significant. The initial absolute neutrophil count had a median of 8.1 (IQR, 5.8–11.5), which decreased to 2.8 (IQR, 2.1–3.9) upon discharge. Again, this decrease, although marked, was not statistically significant. In contrast, the platelet count showed a significant increase, with an initial median of 334 (IQR, 258–451) and a discharge median of 418 (IQR, 312–583; *p* = 0.039).

Among all patients who underwent various radiological tests, three-quarters had abnormal ultrasound findings, a third had abnormal X-ray findings, and all 53 patients who had MRI scans showed abnormalities. CT scans were performed in only three cases, with two revealing abnormal results. While not statistically significant, a higher prevalence of abnormal radiological findings was observed in SA, followed by OM, and then concurrent infections (Table 3).

*S. aureus* was the most frequently isolated organism, detected in 24.1% (15/62) of patients. The incidence of positive blood cultures was highest in concurrent infections at 54.5%, compared with 22% in OM and 9.5% in SA. Positive aspirate cultures were found in 20% of patients with SA, 71.4% with OM, and 55.6% with concurrent infections. Tissue cultures were positive in 33.3%, 60%, and 75% of cases with SA, OM, and concurrent infections, respectively (Tables 3 and 4). In one patient, the aspirate culture gram stain showed gram-positive cocci with failure to grow on culture.

### Antibiotic therapy

Clindamycin was the most frequently prescribed antibiotic, used in 79% of cases. This was followed by the intravenous cephalosporins Ceftriaxone (67.7%) and Cefazolin (40.3%), and the oral cephalosporin cephalexin (25.8%). Other antibiotics were used less frequently: Cloxacillin (16.1%), Vancomycin (9.7%), Rifampicin (6.5%), Piperacillin-Tazobactam (3.2%), Amoxicillin-Clavulanate (3.2%), and Gentamicin (1.6%). Clindamycin was administered to all patients with concurrent infections, 81% of those with SA, and 70% of those with OM (Tables 5 and 6). Significant differences were observed across all routes of antibiotic usage among the groups. The overall median duration for parenteral antibiotics was 13 (IQR, 10–21) days, while the median duration for oral antibiotics was 30 (IQR, 21–42) days. The total median duration of antibiotic therapy was 42 (IQR, 35–56) days. The concurrent infections group had the longest duration of parenteral, oral, and total antibiotic treatments.

### Complications and outcomes

Almost three out of every five patients required surgery (58.1%), with the highest rate observed in the concurrent infections group at 91%, followed by SA at 71.4%, and OM at 36.7% (*p* = 0.002). Apart from surgical interventions, no additional complications were reported in the SA group. In the OM group, there were four cases of abscess or collection and one case of deep vein thrombosis (DVT). The concurrent infections group experienced the highest rate of complications, including abscess/collection (45.5%), septic shock (27.3%), DVT (9.1%), and repeated interventions (27.3%).

## DISCUSSION

In this study, we evaluated 62 children with OAIs and found that those with concurrent infections exhibited more severe disease compared to those with isolated SA or OM. Our findings align with existing literature, illustrating the complexity and varied presentation of OAIs in pediatric patients. Consistent with the work of Yi et al., we identified *S. aureus* as the most frequently isolated pathogen, with a similarity rate of 57.1%, closely matching their reported rate of 51%.^26^

Given the acute and often severe nature of these infections, a significant proportion of children with SA presented with symptoms such as pain, fever, functional limitations, and localized signs.

Our patient cohort consisted of 66.1% males, a proportion closely aligned with the 63% reported by Yi et al. They also reported a median age of 6 years, which is consistent with the 6.03 years median observed in our study. We found that the mean ages for SA and OM were similar (5.4 vs. 5.9 years), with both groups being younger compared to the concurrent infection group (5 vs. 8 years). While Yi et al. noted a significant age difference, with children diagnosed with SA being younger (median 2.9 years) than those with OM (median 8.1 years; *p* ? 0.001), our findings showed comparable ages in the concurrent infection group, consistent with their observations (8.3 vs. 8 years).^26^ The present study findings align with regional studies, such as AlHammadi et al. in Qatar, which primarily focused on OM, reported a male predominance of 62% in OM cases, similar to our 60%. Our mean age of 5.9 years is slightly higher than their 5.7 years. Additionally, Al Zamil et al. in Saudi Arabia found a median age of 3 years and a 75% male predominance. These studies emphasize consistent male predominance and varying age distributions.^25,27^

Compared to the study by Yi et al., our findings are closely matched, with OM being the most common infection type (48.3% vs. 48%), followed by SA (33.The median LOS8% vs. 29%), and concurrent infections (17.7% vs. 23%).^26^ Similarly, Montgomery et al. reported a comparable incidence of concurrent infections at 21.5%, while Trobisch et al. found an incidence of 5%, with literature reviews indicating a variable incidence ranging from 5.5% to 68%. These comparisons underscore the variability in the incidence of concurrent infections across different studies.^28,29^ Similarly, Montgomery et al.

In the study cohort, the lower extremities were predominantly affected, accounting for 80.4% of cases compared to 19.6% for the upper extremities. Specifically, for OM, 79.2% of cases involved the pelvis and lower extremities, which contrasts with the 92% reported by Yi et al. The tibia was identified as the most frequently affected bone in 37.5% of cases, whereas Yi et al. reported the femur as the most affected bone in 24% of their cases, with 25% observed in this current study. Additionally, for SA, the lower extremities were affected in 82.3% of cases, compared to 89% in the study, with the knee being the most commonly affected joint (52.9% vs. 41%).^26^

The median LOS for both OM and SA groups in this study was 11 days. This contrasts with Trobisch et al., who reported a longer LOS for OM compared to SA (10 vs. 7 days, respectively) (1). Both Trobisch et al. and Yi et al. observed a longer LOS for concurrent infections compared to isolated infections, a trend consistent with our findings.^26,29^ However, our study reported a median LOS of 21 days for concurrent infections, which is notably longer than the 8 days reported by Yi et al.^26^ The current study revealed that the majority of patients exhibited elevated initial CRP levels (93.5%) and ESR values (72%), while WBC count was less effective as a diagnostic marker, consistent with findings from Bonhoeffer et al._30_ The median CRP level for SA cases in this study was 102 mg/L, which is higher than the median CRP values reported in other studies, such as 51 mg/L by Yi et al. and 82 mg/L by Trobisch et al.^26,29^ CRP levels were markedly elevated in cases of concurrent infections, with a median of 141 mg/L, comparable to the 137.5 mg/L reported by Yi et al.^26^ This aligns with Carrillo-Marquez et al.’s observation that a CRP level exceeding 100 mg/L is indicative of joint involvement, a finding consistent with our results for both SA and concurrent infections.^31^

In this present study, pathogens were successfully isolated in only 38.8% of cases, which is markedly lower than previous reports. Trobisch et al. and Yi et al. documented pathogen identification rates of 65% and 62%, respectively.^26,29^ Importantly, the highest identification rate was observed in children with concurrent acute OM and SA, reaching 81.8%, which closely resembles the 81% reported by Yi et al. Conversely, the detection rates for isolated OM and SA were substantially lower in our cohort, at 33.3% and 23.8%, compared to 68% for OM and 38% for SA reported by the same study.^26^ Over the past decades, *K. kingae* has emerged as a significant pathogen in pediatric OAIs, with detection rates reaching up to 82% of children under 5 years with SA. However, in our study, no cases of *Kingella* infection were identified. This absence is consistent with our local antibiogram, which also does not report on the presence of *Kingella* in our facilities. It is important to note that our facility, like others in the Middle East, Gulf, and other developing regions, including Africa, relies on a culture-based system rather than polymerase chain reaction (PCR)-based assays used in Europe and the USA. Nonetheless, all cultures in our study were maintained for 7 days before being discarded as negative, ensuring that fastidious, slow-growing organisms like *K. kingae* were not missed.^32,33^

In our study, Clindamycin (79%), Ceftriaxone (67.7%), and Cefazolin (40.3%) were the most frequently prescribed antibiotics for OAIs. Contrarily, a multinational study by Trobisch et al. reported Cefuroxime (44.2%), Clindamycin (28.6%), and Flucloxacillin (25.2%) as the top choices across five countries. Their findings indicated a higher usage of Clindamycin in cases with concurrent OM and SA at 52.6%, compared to OM alone at 33% and SA alone at 20.3%, suggesting a significant risk of MRSA in concurrent cases. Similarly, our study found Clindamycin more frequently used in concurrent OM and SA cases (100%), with lower usage in SA (81%) and OM (70%) cases.

Notably, the current Qatari study revealed a higher prescription rate of Ceftriaxone (67.7%) for OAIs compared with the United Kingdom (25.5%) and the Netherlands (19.3%). In Austria, Clindamycin was predominantly used to treat OM and SA (73.5% and 75%, respectively), followed by Cefuroxime (67.6% and 100%). Similarly, in Germany, Clindamycin was the main treatment for OM and SA (72% and 75%, respectively), with Cefuroxime being less frequently used (56% and 75%). These trends are consistent with our findings, where Clindamycin was the primary treatment for OM and SA (70% and 81%, respectively). However, unlike Austria and Germany, our data showed a higher reliance on Ceftriaxone (60% for OM and 81% for SA), with Cefuroxime being rarely used.^29^ Our antibiotic selection aligns with our institution’s infectious disease guidelines, which are based on both international literature and local antibiogram data.

Our study revealed that all patients exhibited abnormal MRI findings, reinforcing Saavedra-Lozano et al.’s assertion that MRI is the optimal diagnostic tool for OAIs.34 Similarly, Al Zamil et al. reported that all patients with OM demonstrated abnormal MRI results, further supporting the efficacy of MRI in diagnosing these conditions.27 Ultrasound was beneficial in 76.5% of the cases, providing positive findings.^34^ In our cohort, computed tomography was infrequently performed (3 out of 62 cases), consistent with the recommendation by Saavedra-Lozano et al. to reserve its use for scenarios where MRI is unavailable.^34^

In this study, we identified a range of complications that are comparable to those described in the review by Alvares and Mimica. These complications include sepsis or septic shock, DVT, and the need for surgical interventions.^35^

Due to the small sample size, caution is needed when generalizing these findings. While this study offers valuable insights into the clinical and microbiological characteristics of OAIs in children, the limited cases may not reflect broader trends. Diagnosing OAIs remains challenging, often requiring empirical antibiotic therapy while awaiting culture results, which can lead to overtreatment, prolonged intravenous courses, and extended hospital stays. In our setting, a notable proportion of cases received empirical antibiotics despite negative culture results, highlighting the complexity of diagnosis and the need for careful antimicrobial stewardship. Balancing timely treatment with minimizing unnecessary interventions is crucial to optimizing patient outcomes.

While this study offers valuable insights, it is important to acknowledge its limitations. The study utilized a retrospective design, and the data were restricted to pediatric patients within HMC facilities. Additionally, the reliance on a culture-based detection system, which may lack the sensitivity of PCR-based assays, underscores the need for future studies to incorporate PCR to enhance the detection of fastidious or slowly growing organisms like *K. kingae* with greater accuracy. One limitation of this study is the presence of missing data for certain aboratory and imaging parameters. Despite these limitations, this study is significant as it lays the groundwork for future long-term surveillance of OAIs in pediatric patients across tertiary referral hospitals in Qatar. Notably, the governmental pediatric hospitals involved are among the largest in Qatar, emphasizing the importance of this research in addressing a critical gap in the understanding and management of OAIs in this population.

## CONCLUSIONS

OAIs present substantial clinical challenges, marked by complex presentations and diagnostic difficulties. *S. aureus* remains the predominant pathogen, with MRSA accounting for nearly half of the cases. Concurrent infections are linked with more severe complications and prolonged hospitalizations, though they also exhibit the highest rates of positive microbiological results. This study lays the groundwork for future long- term surveillance of OAIs in pediatric patients across tertiary referral hospitals in Qatar. Future research should focus on evaluating the economic burden of OAIs, including hospital-related costs, as well as optimizing management strategies to improve clinical and financial outcomes.

### Acknowledgments

This research was supported by the Medical Research Center (MRC), Hamad Medical Corporation, Doha, Qatar (MRC-01-19-141). We thank our colleagues from the Medical Research Center for sharing their pearls of wisdom with us during this research.

### List of abbreviations

**Table:** 

CRP	C-reactive protein
CT	computed tomography
DVT	deep vein thrombosis
ESR	erythrocyte sedimentation rate
HMC	Hamad Medical Corporation
IQR	interquartile range
*K.*	*kingae Kingella kingae*
LOS	length of stay
MRI	magnetic resonance imaging
MRSA	methicillin-resistant *Staphylococcus aureus*
OAIs	osteoarticular infections
OM	osteomyelitis
PCR	polymerase chain reaction
PCV	pneumococcal conjugate vaccine
SA	septic arthritis
*S. aureus*	*Staphylococcus aureus*
WBC	white blood cell

### Conflict of interest

The authors declare no conflicts of interest.

### Authors’ contributions

Conceptualization: M.E. and S.K. Methodology: T.O. Validation: M.A. Formal analysis: T.O., M.E., E.H., S.S., and K.A. Investigation: E.H. and K.A. Resources: M.E., S.K., and A.K. Data curation: M.E., M.E., E.H., S.S., and K.A. Writing—original draft: M.E. and A.K. Writing—review and editing: M.E., A.K., and A.S. Visualization: M.A.A. Supervision: A.K. All authors have read and agreed to the published version of the manuscript.

### Ethical approval

The study was conducted in accordance with the principles of the Declaration of Helsinki, Good Clinical Practice (GCP), and in compliance with the laws and regulations of the Ministry of Public Health (MOPH) in Qatar. The study received approval from the institutional review board (IRB) committee of the Medical Research Center (MRC), Hamad Medical Corporation, Doha, Qatar, with the number (MRC-01-19-141). The requirement for consent was waived because of the retrospective nature of the study.

### Data availability statement

Upon reasonable request, the primary author can provide the raw data supporting the conclusions of this article.

## Figures and Tables

**Table 1.** Demographics and clinical manifestations.

Demographics	Total (*N* = 62)		SA (*n* = 21)		OM (*n* = 30)		Concurrent infection (*n* = 11)	
	*n*	%	*n*	%	*n*	%	*n*	%	*P* value^†^
**Age, y (mean ± SD)**	6.2 ± 3.5		5.4 ± 3.1		5.9 ± 3.5		8.3 ± 3.8		0.125	
**Sex, male**	41	66.1%	13	61.9%	18	60.0%	10	90.9%	0.158
**National (Qatari)**	23	37.1%	8	38.1%	13	43.3%	2	18.2%	0.334
**Clinical history**									
**Trauma**	21	34.4%	5	23.8%	11	37.9%	5	45.5%	0.407
**Limitation of function**	51	82.3%	18	85.7%	25	83.3%	8	72.7%	NA
**Pain**	55	88.7%	20	95.2%	25	83.3%	10	90.9%	NA
**Fever**	48	77.4%	17	81.0%	22	73.3%	9	81.8%	NA
**Localized signs**	35	58.3%	11	55.0%	19	63.3%	5	50.0%	0.710
**Other symptoms**	9	14.5%	3	14.3%	4	13.3%	2	18.2%	
**LOS**	12	9–20	11	8–15	11	9–16	21	19–40	0.002
**Complications**									
**Surgery**	36	58.1%	15	71.4%	11	36.7%	10	90.9%	0.002
**Abscess/collection**	9	14.5%	0	0.0%	4	13.3%	5	45.5%	NA
**Septic shock**	3	4.8%	0	0.0%	0	0.0%	3	27.3%	NA
**DVT**	2	3.2%	0	0.0%	1	3.3%	1	9.1%	NA
**Repeated interventions**	3	4.8%	0	0.0%	0	0.0%	3	27.3%	NA

Note: ^†^*p* < 0.05.

Abbreviations: DVT: deep venous thrombosis, LOS: length of stay, OM: osteomyelitis, SA: septic arthritis.

NA = tables contained empty cells or more than 30% of cells had expected counts less than 5.

**Table 2.** Infection sites.

Affected site	Total		Septic arthritis		Osteomyelitis		Concurrent infection	
	*n*	%	*n*	%	*n*	%	*n*	%
Knee joint	11	21.6	9	52.9	1	4.2	1	10
Hip joint	11	21.6	5	29.4	2	8.3	4	40
Ankle joint	1	2	0	0	1	4.2	0	0
Femur	6	11.8	0	0	6	25	0	0
Tibia	12	23.5	0	0	9	37.5	3	30
Humerus	4	7.8	0	0	4	16.7	0	0
Elbow joint	6	11.8	3	17.6	1	4.2	2	20
Lower limb	41	80.4	14	82.4	19	79.2	8	80
Upper limb	10	19.6	3	17.6	5	20.8	2	20
Total	51	100	17	100	24	100	10	100

**Table 3.** Laboratory, radiologic findings, and management.

Laboratory	Total (*N* = 62)		Septic arthritis (*n* = 21)		Osteomyelitis (*n* = 30)		Concurrent infection (*n* = 11)		*P* value^†^
	Median	IQR	Median	IQR	Median	IQR	Median	IQR	
**Init WBC**	10.9	9.3–16.4	11.2	10.2–17	10.9	9.1–16.6	10.1	9.1–14.2	0.206
**Dis WBC**	7.9	6.8–9.1	8.5	7.1–11.9	7.9	7–8.6	6.8	6–8.1	0.061
**Init ANC**	8.1	5.8–11.5	7.7	5.5–14.2	8.2	5.8–11	8.2	7.2–10.1	0.976
**Dis ANC**	2.8	2.1–3.9	3.7	2.4–5.4	2.1	1.7–2.8	3.1	2.7–3.6	0.006
**Init Hgb**	11.5	10.4–12	11.1	10.4–12.4	11.5	10.5–12.2	11.8	10.4–11.9	0.940
**Dis Hgb**	11.3	10–12	11.0	9.4–11.8	11.4	10.5–12.2	11.1	9.7–11.4	0.252
**Init PLT**	334	258–451	334	299–473	344	270–497	236	213–313	0.039
**Dis PLT**	418	312–583	483	334–587	412	305–582	364	283–610	0.768
**Init CRP**	84	33–134	102	52–134	54	22–111	141	44–249	0.056
**Dis CRP**	5	3–11	7	3–22	5	3–10	5	3–14	0.459
**Init ESR**	34	9–52	40	10–81	29	5–50	35	16–52	0.442
**Dis ESR**	34	19–46	35	19.5–56	30	16–46	40	27–43	0.721
**Init PCT**	0.34	0.07–2.7	0.07	0.06–0.08	0.8	0.07–2.7	62.6	1.02–62.64	0.032
**Dis PCT**	0.09	0.05–0.12	0.06	0.05–0.11	0.12	0.04–0.17	1.08	0.06–2.09	0.486
**Positive cultures**									
**Blood**	14/59	23.7%	2/21	9.5%	6/27	22.2%	6/11	54.5%	
**Aspirate**	13/31	41.9%	3/15	20.0%	5/7	71.4%	5/9	55.6%	
**Tissue**	15/27	55.6%	3/9	33.3%	6/10	60.0%	6/8	75.0%	
**Radiological findings**									
**US**	39	76.5%	17	89.5%	16	69.6%	6	66.7%	
**X-ray**	19	35.2%	7	43.8%	9	33.3%	3	27.3%	0.651
**MRI**	53	100%	13	100%	29	100%	11	100%	
**CT**	2	66.7%	1	100%	1	50%	-	-	
**Antibiotic duration**									
**Intravenous**	13	10–21	13	10–15	11	9–15	23	19–35	<0.001
**Oral**	30	21–42	21	14–28	34	30–49	42	19–63	<0.001
**Total duration**	42	35–56	29	28–42	43	42–60	57	42–90	<0.001

Note: ^†^*p* < 0.05.

Abbreviations: ANC: absolute neutrophil count, CRP: C-reactive protein, CT: computed tomography, Dis: discharge, ESR: erythrocyte sedimentation rate, Hgb: hemoglobin, Init: initial, MRI: magnetic resonance imaging, PCT: procalcitonin, PLT: platelets, US: ultrasound, WBC: white blood cells.

**Table 4.** Isolated organism per diagnosis.

Isolated organism (from blood or site cultures)	All	Septic arthritis	Osteomyelitis	Concurrent infection
Total	62	21 (33.88%)	30 (48.38)	11 (17.74)
Isolated	24 (38.8%)	5 (23.8%)	10 (33.3%)	9 (81.8%)
None	38 (61.2%)	16 (76.2%)	20 (66.7%)	2 (18.1%)
MSSA	8 (12.9%)	1 (4.76%)	5 (16.6%)	2 (18.1%)
MRSA	7 (11.2%)	1 (4.76%)	2 (6.6%)	4 (36.4%)
*S. Pyogenes*	6 (9.6%)	2 (9.52%)	2 (6.6%)	2 (18.1%)
*S. Pneumoniae*	1 (1.6%)	1 (4.76%)	0	0
*P. Aeruginosa*	1 (1.6%)	0	1 (3.3%)	0
Gram-positive Cocci	1 (1.6%)	0	0	1 (9%)

Note: MSSA: methicillin-sensitive *Staphylococcus aureus*, MRSA: methicillin-resistant *Staphylococcus aureus*, *P. Aeruginosa*: *Pseudomonas Aeruginosa*, *S. Pyogenes*: *Streptococcus Pyogenes*, *S. Pneumoniae*: *Streptococcus Pneumoniae*.

**Table 5.** Antimicrobial selection.

	Overall (*N* = 62)	Septic arthritis (*n* = 21)	Osteomyelitis (*n* = 30)	Concurrent infection (*n* = 11)
**Clindamycin**	**79.0%**	**81.0%**	**70.0%**	**100.0%**
**Ceftriaxone**	67.7%	81.0%	60.0%	63.6%
**Cefazolin**	40.3%	33.3%	46.7%	36.4%
**Cloxacillin**	16.1%	9.5%	20.0%	18.2%
**Vancomycin**	9.7%	4.8%	-	45.5%
**Gentamicin**	1.6%	-	-	9.1%
**Piperacillin/Tazobactam**	3.2%	4.8%	3.3%	
**Rifampicin**	6.5%	4.8%	3.3%	18.2%
**Cephalexin**	25.8%	9.5%	36.7%	27.3%
**Amoxicillin/Clavulanate**	3.2%	-	3.3%	9.1%

**Table 6.** Antimicrobial susceptibility.

Microorganism	Sensitive	Resistant
MRSA (seven cases)	Clindamycin, Gentamicin, Sulfamethoxazole/Trimethoprim, Vancomycin	Cloxacillin, Penicillin
	Clindamycin, Rifampin, Teicoplanin, Sulfamethoxazole/Trimethoprim, Vancomycin	Cloxacillin, Cefazolin
	Linezolid, Rifampin, Teicoplanin, Sulfamethoxazole/Trimethoprim, Vancomycin	Cefazolin, Clindamycin, Cloxacillin, Penicillin
	Vancomycin, Rifampin, Gentamicin	Cefazolin, Clindamycin, Cloxacillin, Erythromycin, Tetracycline, Sulfamethoxazole/Trimethoprim
	Vancomycin, Rifampin, Gentamicin, Ciprofloxacin, Teicoplanin, Tetracycline, Sulfamethoxazole/Trimethoprim	Cefazolin, Clindamycin, Cloxacillin, Erythromycin, Penicillin
	Clindamycin, Rifampin, Vancomycin, Teicoplanin, Sulfamethoxazole/Trimethoprim	Cefazolin, Gentamicin, Cloxacillin, Penicillin
	Vancomycin, Ciprofloxacin, Teicoplanin, Clindamycin, Rifampin, Gentamycin	Cloxacillin, Penicillin, Cefazolin, Sulfamethoxazole/Trimethoprim
MSSA (eight cases)	Cefazolin, Clindamycin, Cloxacillin, Gentamicin	Penicillin
	Cefazolin, Clindamycin, Cloxacillin, Gentamicin	Erythromycin
	Cefazolin, Clindamycin, Cloxacillin, Erythromycin	None reported
	Cefazolin, Clindamycin, Cloxacillin, Gentamicin	None reported
	Cefazolin, Clindamycin, Cloxacillin, Gentamicin, Rifampin	Penicillin, Sulfamethoxazole/Trimethoprim
	Oxacillin, Clindamycin, Erythromycin	Penicillin, Sulfamethoxazole/Trimethoprim
	Cefazolin, Clindamycin, Cloxacillin, Erythromycin, Oxacillin	Penicillin, Ampicillin
	Cefazolin, Clindamycin, Cloxacillin, Gentamicin, Rifampin, Oxacillin	Penicillin
*S. Pyogenes* (five cases)	Ceftriaxone, Penicillin	None reported
	Ceftriaxone, Penicillin	None reported
	Ceftriaxone, Penicillin, Clindamycin	None reported
	Ceftriaxone, Penicillin, Clindamycin	None reported
	Penicillin	None reported
*Streptococcus pneumoniae*	Ceftriaxone, Meropenem	Penicillin
*Pseudomonas Aeruginosa*	Cefepime, Genta, Tazocin	None reported

Note: MSSA: methicillin-sensitive *Staphylococcus aureus*, MRSA: methicillin-resistant *Staphylococcus aureus, S. Pyogenes: Streptococcus Pyogenes*.
